# 4-Week Comparative Outcomes of Standard Physiotherapy, Balneotherapy Plus Physiotherapy and Dextrose Prolotherapy in Patients with Low Back Pain: A Non-Randomized Study

**DOI:** 10.3390/medicina62040623

**Published:** 2026-03-25

**Authors:** Stelian Ilie Mociu, Elena Valentina Ionescu, Andreea-Bianca Uzun, Nicolae Ciufu, Alexandra Ecaterina Ciota, Teodora Elena Iliescu, Ioan Calatoiu, Lucian Cristian Petcu, Madalina-Gabriela Iliescu

**Affiliations:** 1 Doctoral School of Medicine, Ovidius University of Constanta, 1 University Alley, Campus—Corp B, 900470 Constanta, Romania; stelian.mociu@365.univ-ovidius.ro (S.I.M.); bianca.uzun@365.univ-ovidius.ro (A.-B.U.); alexandra.ciota@365.univ-ovidius.ro (A.E.C.); lucian-cristian.petcu@365.univ-ovidius.ro (L.C.P.); madalina.iliescu@365.univ-ovidius.ro (M.-G.I.); 2 Ovidius Clinical Hospital, DN2A km 202 + 880, Ovidiu, 905900 Constanta, Romania; nicolae.ciufu@365.univ-ovidius.ro; 3 Department of Palliative Care, Faculty of Medicine, Ovidius University of Constanta, 1 University Alley, Campus—Corp B, 900470 Constanta, Romania; 4 Department of Physical Medicine and Rehabilitation, Faculty of Medicine, Ovidius University of Constanta, 1 University Alley, Campus—Corp B, 900470 Constanta, Romania; 5 Balneal and Rehabilitation Sanatorium of Techirghiol, 34-40, Dr. Victor Climescu Street, 906100 Techirghiol, Romania; 6 Department of General Surgery, Faculty of Medicine, Ovidius University of Constanta, 1 University Alley, Campus—Corp B, 900470 Constanta, Romania; 7 Faculty of Medicine, Carol Davila University of Medicine and Pharmacy, Dionisie Lupu Street, No. 37, Sector 2, 020021 Bucharest, Romania; teodora-elena.iliescu25@rez.umfcd.ro (T.E.I.); ioan.calatoiu25@rez.umfcd.ro (I.C.)

**Keywords:** low back pain, physiotherapy, balneotherapy, prolotherapy, pain assessment, functional disability, quality of life

## Abstract

*Background and Objectives*: Low back pain (LBP) represents a major cause of disability worldwide, with a significant impact on quality of life and functional capacity. Standard physiotherapy is widely used for LBP, whereas comparative short-term evidence on balneotherapy and prolotherapy remains limited. This study aimed to compare clinical outcomes across therapeutic approaches in patients with LBP. *Materials and Methods:* This non-randomized, three-group interventional cohort study included adult patients diagnosed with LBP lasting more than three months and presenting a baseline Visual Analog Scale (VAS) score ≥ 4. 84 patients were allocated to one of three treatment groups: standard physiotherapy (CG) (25 patients), balneotherapy plus physiotherapy (BG) (28 patients), or prolotherapy (PG) (31 patients). Outcome measures included pain intensity, functional disability, lumbar mobility, quality of life, and psychological status. This clinical study has been officially registered on ClinicalTrials.gov under the identifier NCT07399145. *Results:* Statistical analyses were performed to assess within- and between-group differences over time. At the 4-week follow-up, all three treatment groups showed significant improvements compared to baseline in pain intensity (VAS; *p* < 0.001 for PG, BG, and CG) and lumbar mobility (Schober test; *p* < 0.001 for PG, BG, and CG), and functional disability (RMDQ; *p* < 0.001 for PG, *p* = 0.027 for BG, and *p* = 0.016 for CG). Between-group analyses at T1 revealed significant differences for RMDQ, ODI, Schober, and EQ-5D-5L. In contrast, no significant intergroup differences were observed for VAS, PPT-RS, PPT-LS, total PPT, HADS-A, HADS-D, BDI-II, or PHQ-9. *Conclusions:* All evaluated interventions improved clinical, functional, and psychological outcomes in patients with LBP. Prolotherapy showed a pattern of more consistent improvements, particularly in disability, spinal mobility, and health-related quality of life. These findings should be interpreted cautiously, given the non-randomized design and baseline differences between groups. Further randomized studies with larger samples and longer follow-up are warranted to confirm these results.

## 1. Introduction

Low back pain (LBP) is among the most common causes of long-term disability in adults and represents a major burden for healthcare systems worldwide [1,2]. Beyond its high prevalence, LBP is characterized by a complex, heterogeneous etiology, with mechanical, degenerative, inflammatory, and psychosocial factors often coexisting [3,4]. As a result, symptom persistence and functional limitation vary widely between patients, making therapeutic decisions particularly challenging in daily clinical practice [5].

Psychosocial factors represent major determinants of the course of LBP. Elevated stress levels, lack of social support, job dissatisfaction, and affective disorders are associated with an increased risk of pain chronicity and a poorer therapeutic response [6]. Longitudinal studies have shown that depression and anxiety significantly influence pain perception and functional disability, independently of the severity of structural lesions [7,8].

Conservative management remains the first-line approach for LBP, with standard physiotherapy playing a central role in rehabilitation programs. LBP is a highly prevalent musculoskeletal condition with a multifactorial etiology, commonly managed through standard physiotherapy approaches that include individualized kinesitherapy programs [9,10], electrotherapy modalities such as interferential currents [11] and transcutaneous electrical nerve stimulation [12], as well as adjunctive physical agents including ultrasound [13], laser therapy [14], and therapeutic massage [15,16], aiming to reduce pain, improve functional capacity, and enhance quality of life. Conventional physiotherapy constitutes a standardized and widely accessible conservative treatment strategy centered on exercise-based lumbar stabilization, supported by electrotherapy and manual interventions, and is commonly recommended as first-line management for non-specific LBP [11,13,14].

Despite the widespread use of conventional physiotherapy, complementary therapeutic options are increasingly integrated into the management of LBP. Balneotherapy, often administered alongside physiotherapy, is frequently employed in patients with chronic musculoskeletal disorders and is thought to exert beneficial effects through thermal and mechanical mechanisms [17,18,19]. Balneotherapy combines natural therapeutic factors, such as mineral-rich saline water and sapropelic mud, with structured rehabilitation, and is traditionally indicated for patients with chronic musculoskeletal conditions who may benefit from multimodal, spa-based interventions targeting pain modulation, circulation, and tissue recovery [18,19].

Moreover, the mineral waters of Techirghiol are rich in sodium chloride, bromide, iodide, and essential trace elements, contributing to their therapeutic effects. The Techirghiol therapeutic mud exhibits well-documented anti-inflammatory and analgesic properties and is widely used in the treatment of rheumatic, neurological, immune, and chronic respiratory conditions [19,20]. In parallel, prolotherapy has emerged as a regenerative, injection-based technique that stimulates local tissue repair and modulates pain perception [21]. Although both approaches are commonly used in clinical practice, direct comparative evidence with standard physiotherapy remains limited.

Prolotherapy, a minimally invasive interventional method that involves injecting irritant solutions (usually hypertonic glucose) into the affected structures, has gained ground in recent years as an option for patients with chronic low back pain associated with ligamentous instability or soft tissue injuries. Studies suggest that it may lead to significant pain reduction and functional improvement, especially when combined with lumbopelvic stabilization exercises [22]. According to the analysis conducted by Hauser and colleagues, the method involves injecting solutions with an irritant or regenerative role into the affected regions to stimulate the body’s natural tissue repair processes and restore stability to the lumbar segment [23]. The mechanism by which this method acts involves stimulating growth factors and fibroblasts at the injection site, leading to strengthening of ligamentous attachments and improved structural integrity of the treated area [24]. May and colleagues highlight that the success of this procedure depends significantly on a thorough clinical evaluation, an in-depth knowledge of the relevant anatomy, and a precise injection technique [25]. The clinical indications for prolotherapy are varied and are not limited to the lumbar region. In addition to pain associated with disc degeneration, facet joint osteoarthritis, or sacroiliac joint dysfunction, prolotherapy is also used in cases of: lateral epicondylitis, rotator cuff tendinopathy, plantar fasciitis, Achilles tendinopathy, osteoarthritis, and temporomandibular joint instability [26]. The effectiveness of prolotherapy in cases of non-specific low back pain has been the subject of numerous clinical studies and systematic reviews, with generally optimistic results, particularly when this technique is applied in combination with other forms of regenerative treatments [27,28,29,30,31].

Most available studies focus on single treatment modalities or evaluate outcomes under controlled conditions. However, in real-world rehabilitation settings, treatment allocation is rarely randomized and is often influenced by symptom severity, patient preference, and the availability of therapeutic resources. Comparative data reflecting this clinical reality remain limited.

Therefore, the present study aimed to compare short-term clinical outcomes of standard physiotherapy, balneotherapy combined with physiotherapy, and prolotherapy in patients with LBP, using validated measures of pain intensity, functional disability, lumbar mobility, quality of life, and psychological status, assessed at baseline and at a 4-week follow-up.

## 2. Materials and Methods

### 2.1. Patient Selection

A total of 84 patients were included in the study and allocated to three groups: the standard physiotherapy group (CG, n = 25), the balneotherapy plus physiotherapy group (BG, n = 28), and the dextrose prolotherapy group (PG, n = 31).

As no formal a priori sample size calculation was performed, the study should be considered exploratory in nature, and the findings should therefore be interpreted as preliminary and hypothesis-generating.

**Figure 1:**
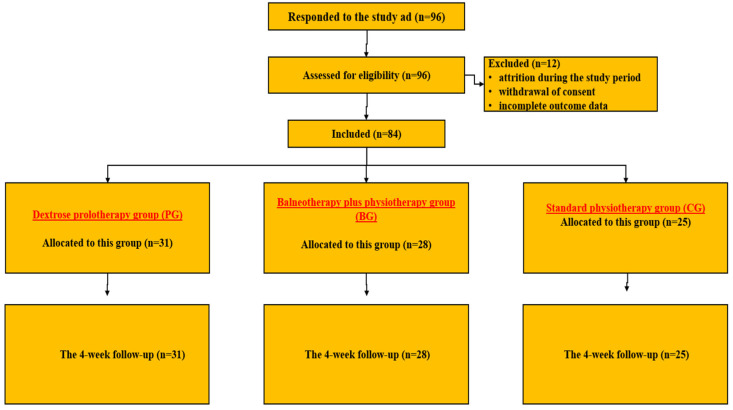
The patient selection process.

In Figure 1, the patient selection process is represented in the form of a flow diagram.

The study was conducted as a non-randomized controlled pragmatic study (quasi-experimental design), with allocation to interventions based on clinical indication. This design reflects real-world clinical practice while acknowledging the absence of randomization.

This prospective, multicenter, interventional, controlled, comparative clinical study (Clinical Trials.gov number: NCT07399145) included adult patients diagnosed with LBP who were consecutively recruited from participating rehabilitation and balneotherapy centers between May and December 2025.

This study was retrospectively registered on ClinicalTrials.gov during the preparation of the manuscript for publication. Ethical approval was obtained before study initiation from the Ethics Committees of the Balneal and Rehabilitation Sanatorium of Techirghiol (approval number 06/25 March 2025) and Ovidius Clinical Hospital (approval number 76/25 March 2025). The study protocol, eligibility criteria, interventions, and outcome measures were predefined before patient recruitment and were not modified after registration.

Patient enrollment was conducted across multiple centers to reflect routine clinical practice and enhance the external validity of the study findings. Patients were recruited from two clinical centers. Participants allocated to the PG and the CG were treated at Ovidius Clinical Hospital, Romania. At the same time, patients included in the BG underwent treatment at the Balneal and Rehabilitation Sanatorium of Techirghiol, Romania.

Participants were enrolled consecutively at the participating centers according to treatment availability and referral pathways within the pragmatic multicenter design. The three groups were initially comparable in size at enrollment. However, differences in the final sample sizes resulted from attrition during the study period, withdrawal of consent, and incomplete outcome data.

Eligible participants were allocated to one of three treatment groups in a non-randomized manner. Treatment allocation was based on clinical indication, patient eligibility for specific interventions, and the availability of therapeutic resources at each participating center, particularly for balneotherapy, which is limited to specialized facilities. Randomization was not feasible due to the multicentric design of the study and the limited access to balneotherapy services, which cannot be offered uniformly across all centers.

This approach was intentionally chosen to mirror real-world rehabilitation settings, where therapeutic decisions are commonly influenced by logistical constraints and patient-specific factors. Although the prolotherapy and control groups were managed at the same center, allocation was determined by treatment eligibility and patient consent for the interventional procedure rather than by random assignment. No post-enrollment reallocation occurred.

Inclusion Criteria:

• Clinical diagnosis of LBP lasting more than three months, confirmed by lumbar radiography or magnetic resonance imaging;
• Pain intensity of at least 4 on the Visual Analog Scale (VAS);
• Age between 18 and 70 years;
• Patients who can comply with the study protocol and scheduled evaluations;
• Patients who provided written informed consent before participation.

Exclusion Criteria:

• Lumbar disc herniation with surgical indication or severe radiculopathy;
• Active infections;
• Systemic inflammatory or autoimmune diseases;
• A history of allergy to substances used in dextrose prolotherapy;
• Contraindications to balneotherapy, such as severe cardiovascular disease, renal insufficiency, or severe dermatological conditions;
• Prior prolotherapy or balneotherapy within six months before enrollment;
• Decompensated chronic cardiovascular, hepatic, renal, respiratory, or neurological diseases at the time of inclusion;
• Pregnancy;
• Breastfeeding;
• Active neoplastic disease;
• Decompensated psychiatric disorders;
• Allergies to natural therapeutic factors that could lead to treatment interruption;
• Refusal to participate in the study.

### 2.2. Ethical Issues

The study was approved by the Ethics Committee of the Balneal and Rehabilitation Sanatorium of Techirghiol (number 06/25 March 2025), and Ovidius Clinical Hospital (number 76/25 March 2025), and all patients signed informed consent. All procedures were conducted in accordance with the ethical standards of the Declaration of Helsinki.

### 2.3. Study Design

All participants were instructed to maintain their usual daily activities and were advised not to initiate any new treatments targeting low back pain throughout the follow-up period. No co-interventions were permitted.

**Figure 2:**
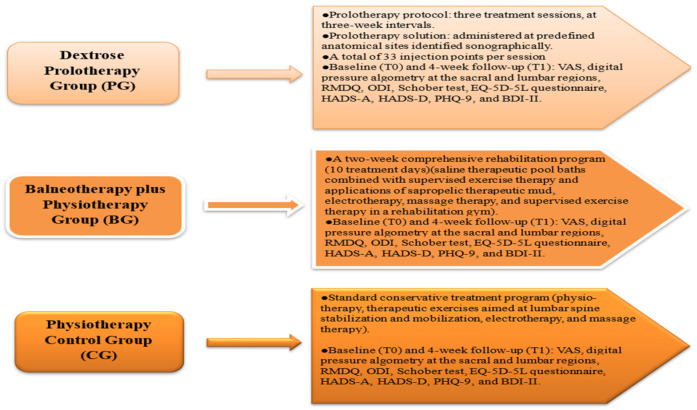
Study design.

Study design and applied therapies are summarized in Figure 2.

#### 2.3.1. Dextrose Prolotherapy Group (PG)

The prolotherapy protocol consisted of three treatment sessions, performed at three-week intervals. All procedures were carried out by a single investigator, an anesthesiology and intensive care physician with experience in ultrasound-guided interventional techniques, to minimize operator-related variability.

All injections were performed bilaterally under real-time ultrasound guidance, using a BK Medical Flex Focus 800 system equipped with a convex transducer. Ultrasound imaging was used to ensure accurate identification of anatomical landmarks and target structures before injection.

**Figure 3:**
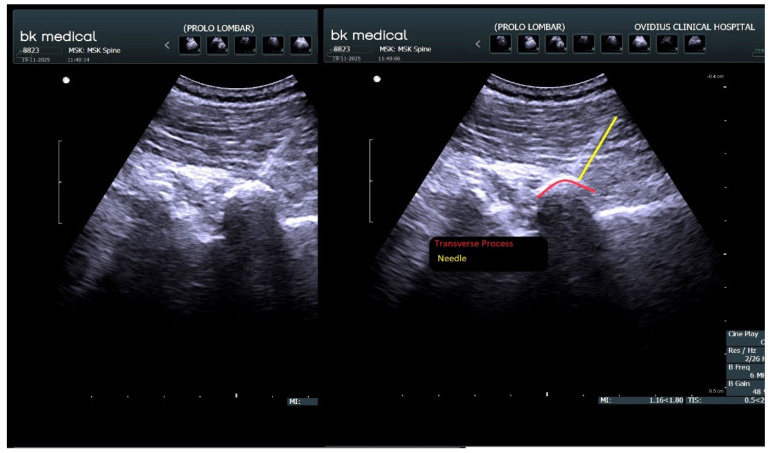
Ultrasound image showing ultrasound-guided needle placement adjacent to the lumbar transverse process during prolotherapy. Source: Personal archive.

The prolotherapy solution was administered at predefined anatomical sites identified sonographically, including one injection point corresponding to the piriformis muscle, five points along the iliac crests corresponding to ligamentous insertions, three points at the sacral level, and five points at the level of the lumbar transverse processes targeting paravertebral ligamentous insertions. In addition, five unilateral injections were performed at the level of the lumbar spinous processes (Figure 3). In total, 33 injection points were used per session. A volume of 1 mL of prolotherapy solution was administered at each injection site [32].

The injected solution consisted of a 1:1 dilution of 33% dextrose and 1% lidocaine, resulting in a final dextrose concentration of 16.5%. This formulation was used consistently across all injection sites to ensure standardized delivery and reproducible therapeutic effect [26].

All infiltration procedures were performed under strict aseptic conditions [33]. The interventions were conducted under intravenous analgosedation, using a single-dose regimen of midazolam (2 mg), propofol (approximately 60 mg), and fentanyl (0.1 mg), administered to ensure patient comfort and procedural compliance. Continuous monitoring of vital parameters, including blood pressure, heart rate, and peripheral oxygen saturation, was maintained throughout the procedure and during the immediate post-procedural period, with supplemental oxygen provided as needed [34,35,36].

Following each treatment session, patients were clinically monitored for the detection of any immediate adverse reactions or procedural complications [37].

Patients in the PG did not receive any additional physiotherapy or other structured therapeutic interventions during the study period.

#### 2.3.2. Balneotherapy Plus Physiotherapy Group (BG)

Patients in the BG underwent a two-week comprehensive rehabilitation program (10 treatment days) at the Balneal and Rehabilitation Sanatorium of Techirghiol, Romania, based on natural therapeutic factors specific to the Techirghiol area. The program included saline therapeutic pool baths combined with supervised exercise therapy and applications of sapropelic therapeutic mud, with sessions performed daily, five days per week, under medical supervision [20,38,39,40].

In addition to balneological procedures, patients received daily electrotherapy according to a standardized protocol. Lumbar magnetotherapy was applied for 20 min per session [41], followed by interferential current therapy using two successive modes: a manual mode at 80 Hz for 10 min and a spectrum mode with variable frequencies between 0 and 100 Hz for an additional 10 min [42]. Therapeutic ultrasound was applied at the lumbar level using a frequency of 1 MHz and an intensity of 0.8 W/cm^2^ [43]. All electrotherapy procedures were supervised by specialized personnel and adjusted based on individual tolerance.

The rehabilitation program was complemented by daily massage therapy and supervised exercise therapy in a rehabilitation gym. Massage therapy targeted the lumbar and paravertebral regions using classical techniques, while exercise sessions lasted approximately 30 min and focused on strengthening the paravertebral and abdominal muscles, improving lumbar mobility, and enhancing segmental stability and postural control [16,44,45].

#### 2.3.3. Standard Physiotherapy Group (CG)

Patients in the CG were evaluated and treated at the Ovidius Clinical Hospital in Romania and followed a standard conservative treatment program (10 treatment days). The intervention included physiotherapy, therapeutic exercises aimed at lumbar spine stabilization and mobilization, electrotherapy, and massage therapy, without the use of prolotherapy or balneotherapy, according to the same methodological principles described above [38,40,41,42,43,44].

Importantly, the physiotherapy protocol administered in the CG was identical in structure, duration, frequency, and therapeutic modalities to that provided in the BG, with the sole exception of the saline therapeutic pool baths and sapropelic mud applications.

### 2.4. Outcome Assessment

Baseline assessments (T0), performed before treatment initiation, included demographic and clinical characteristics (age, sex, body mass index, living environment, and physical activity level). Pain intensity was assessed using VAS [46], while pressure pain sensitivity was evaluated by digital pressure algometry at the sacral and lumbar regions [47]. Functional disability was assessed using the Roland–Morris Disability Questionnaire (RMDQ) [48] and the Oswestry Disability Index (ODI) [49]. Lumbar spine mobility was evaluated using the Schober test [50]. Health-related quality of life was assessed using the EuroQol 5-Dimension 5-Level questionnaire (EQ-5D-5L questionnaire) [51]. Psychological status was evaluated using the Hospital Anxiety and Depression Scale (HADS-A and HADS-D), the Patient Health Questionnaire-9 (PHQ-9), and the Beck Depression Inventory-II (BDI-II) [52,53,54].

Pain intensity was assessed using VAS, a validated and sensitive measure of subjective pain perception in LBP [46]. Functional disability was evaluated using both RMDQ and ODI, as these instruments capture complementary aspects of back-specific disability. RMDQ is particularly sensitive to mild-to-moderate disability, whereas ODI provides a broader assessment of functional impairment in daily activities [48,49].

Lumbar mobility was assessed using the Schober test, an objective clinical measure of lumbar flexibility [50]. Health-related quality of life was evaluated using EQ-5D-5L, a standardized instrument allowing multidimensional assessment of general health status [51].

Psychological status was assessed using HADS-A, HADS-D, PHQ-9, and BDI-II to capture complementary dimensions of emotional distress [52,53,54]. HADS was selected for its suitability in medical populations, minimizing overlap with somatic symptoms [52]. PHQ-9 was included as a brief diagnostic and statistical manual of mental disorders -aligned screening tool for depressive symptoms [53], while BDI-II provided a more comprehensive evaluation of depressive severity, including cognitive and affective domains [54]. The use of multiple validated measures allowed for a multidimensional assessment of psychological outcomes and enhanced the robustness of the findings.

Follow-up assessments were conducted at 4 weeks after treatment completion (T1). The same outcome measures were reassessed at follow-up, including VAS, pressure pain thresholds (PPT), RMDQ, ODI, Schober test, EQ-5D-5L, HADS-A, HADS-D, PHQ-9, and BDI-II, using identical standardized instruments and evaluation procedures as at baseline.

### 2.5. Statistical Analysis

Statistical analyses were conducted using IBM SPSS Statistics version 25. Because the normality assumption assessed with the Shapiro–Wilk test was not met for the numerical variables in the CG, PG, and BG groups (*p* < 0.05), a non-parametric analytical approach was applied. The Kruskal–Wallis test was used to examine differences among the three groups in the distribution of the analyzed scores at both time points (T0 and T1), followed by post hoc pairwise comparisons with Bonferroni correction to minimize the risk of Type I errors. Additionally, the Wilcoxon signed rank test was performed within each group to compare changes between the initial (T0) and final (T1) assessments [55].

### 2.6. Bias Control and Methodological Considerations

Given the non-randomized pragmatic design, full elimination of bias was not feasible. However, several measures were implemented to minimize potential sources of bias. Standardized treatment protocols were applied within each group, and all participants underwent identical assessment procedures at baseline and follow-up. Validated and widely used outcome instruments were employed to enhance measurement reliability. Data collection followed a predefined protocol to ensure procedural consistency across centers. Although allocation was based on clinical indication and logistical considerations, no post-enrollment reassignment occurred. The same statistical framework was applied uniformly across groups to ensure comparability of analyses [56,57].

## 3. Results

Baseline demographic characteristics of the patients are presented in Table 1. A total of 84 patients were included in the analysis. A statistically significant age difference was observed between the study groups (*p* < 0.001), with patients in the balneotherapy plus physiotherapy group being older compared with those in the control and prolotherapy groups. No significant differences were found between groups with respect to sex distribution, body mass index categories, living environment, or physical activity level (*p* > 0.05 for all).

The Kruskal–Wallis test indicated the presence of significant differences among the three groups regarding the distribution of VAS scores at T0 (H = 8.442, df = 2, *p* = 0.015). Post hoc tests with Bonferroni correction revealed a significant difference only between the BG and PG groups (test statistic = 16.289, Z = 2.594, p_adj._ = 0.028). No significant differences were found between CG and PG (test statistic = 15.248, Z = 2.355, p_adj._ = 0.056) or between BG and CG (test statistic = −1.041, Z = −0.157, p_adj._ = 1.000). At T1, the Kruskal–Wallis test did not reveal significant differences among the three groups in terms of the distribution of VAS scores (H = 0.595, df = 2, *p* = 0.743). The Wilcoxon test applied separately within each group between baseline (T0) and the final time point (T1) showed significant differences in the distribution of VAS scores, suggesting significant improvements in perceived pain intensity in all three groups: PG (Z = −4.880, *p* < 0.001), BG (Z = −3.990, *p* < 0.001), and CG (Z = −4.315, *p* < 0.001) (Table 2).

For PPT RS, no statistically significant changes were found in any group: PG (Z = 0.803, *p* = 0.422), BG (Z = 0.023, *p* = 0.982), and CG (Z = −1.184, *p* = 0.236). Kruskal–Wallis test revealed no significant differences at T0 (H = 1.197, df = 2, *p* = 0.550) or T1 (H = 0.896, df = 2, *p* = 0.639) (Table 2).

For PPT LS, significant improvement was observed only in PG (Z = 2.273, *p* = 0.023), while BG (Z = 0.068, *p* = 0.946) and CG (Z = 0.632, *p* = 0.527) showed no changes. Kruskal–Wallis comparisons showed no significant differences at T0 (H = 2.155, df = 2, *p* = 0.341) or T1 (H = 1.241, df = 2, *p* = 0.538) (Table 2).

Regarding total PPT, PG did not show significant improvement (Z = 1.254, *p* = 0.210), and no significant differences were observed at either T0 (H = 0.998, df = 2, *p* = 0.607) or T1 (H = 0.019, df = 2, *p* = 0.991) (Table 2).

Regarding HADS-D, PG showed a significant decrease (Z = −2.716, *p* = 0.007), as did CG (Z = −2.724, *p* = 0.006). The reduction in BG was not statistically significant (Z = −1.880, *p* = 0.060). No significant between-group differences were detected at T0 (H = 2.614, df = 2, *p* = 0.271) or T1 (H = 3.164, df = 2, *p* = 0.206). PHQ-9 scores significantly decreased in all three groups: PG (Z = −3.279, *p* = 0.001), BG (Z = −3.126, *p* = 0.002), and CG (Z = −2.530, *p* = 0.011). However, Kruskal–Wallis tests showed no significant differences between groups at either T0 (H = 3.352, df = 2, *p* = 0.187) or T1 (H = 1.748, df = 2, *p* = 0.417) (Table 3).

BDI-II scores also decreased significantly in all groups: PG (Z = −3.348, *p* < 0.001), BG (Z = −2.971, *p* = 0.003), and CG (Z = −2.850, *p* = 0.004). No statistically significant differences between groups were observed at T0 (H = 2.862, df = 2, *p* = 0.239) or T1 (H = 2.685, df = 2, *p* = 0.261) (Table 3).

Kruskal–Wallis tests did not show significant differences between groups for HADS-D scores at either T0 (H = 2.614, df = 2, *p* = 0.271) or T1 (H = 3.164, df = 2, *p* = 0.206), suggesting similar distributions of depressive symptoms. Intra-group analysis revealed significant reductions in scores for PG (Z = −2.716, *p* = 0.007) and CG (Z = −2.724, *p* = 0.006), but not for BG (Z = −1.880, *p* = 0.060). Thus, symptom improvement was statistically significant only in PG and CG. No significant between-group differences were observed for PHQ-9 scores at either T0 (H = 3.352, *p* = 0.187) or T1 (H = 1.748, *p* = 0.417), indicating comparable distributions. However, all three groups showed significant within-group reductions: PG (Z = −3.279, *p* = 0.001), BG (Z = −3.126, *p* = 0.002), and CG (Z = −2.530, *p* = 0.011), suggesting a general decrease in self-reported depressive symptoms. BDI-II scores did not differ significantly between groups (T0: H = 2.862, *p* = 0.239; T1: H = 2.685, *p* = 0.261). In contrast, significant intra-group reductions were found in all three groups: PG (Z = −3.348, *p* < 0.001), BG (Z = −2.971, *p* = 0.003), and CG (Z = −2.850, *p* = 0.004), indicating a meaningful decrease in depressive symptoms across the entire sample (Table 3).

The Kruskal–Wallis analysis indicated significant differences among the three groups regarding the distribution of RMDQ scores at T0 (H = 10.339, df = 2, *p* = 0.006). Post hoc tests with Bonferroni correction confirmed the presence of significant between-group differences. A significant difference was observed between CG and PG (test statistic = 18.189, Z = 2.790, p_adj._ = 0.016), and a comparable significant difference was found between CG and BG (test statistic = 19.042, Z = 2.853, p_adj._ = 0.013). The PG–BG comparison did not indicate a significant difference (test statistic = −0.853, Z = −0.135, p_adj._ = 1.000). These findings suggest that the level of functional disability, as measured by the RMDQ at baseline (T0), differed significantly depending on group allocation, particularly in comparisons involving the CG group, where scores appeared to be higher. The Kruskal–Wallis analysis also revealed significant differences among the three groups in the distribution of RMDQ scores at T1 (H = 7.900, df = 2, *p* = 0.019). Post hoc tests with Bonferroni correction identified a significant difference between CG and BG (test statistic = 18.692, Z = 2.810, p_adj._ = 0.015). A moderate difference was observed between CG and PG (test statistic = 10.105, Z = 1.555); however, the adjusted *p*-value (0.360) did not reach statistical significance. The PG–BG comparison did not indicate a significant difference (test statistic = −8.587, Z = −1.363, p_adj._ = 0.519). These results suggest that functional disability at T1 differed significantly between groups, particularly in the CG–BG comparison, where the difference was statistically robust. The remaining pairwise comparisons were not significant after adjustment for multiple testing. The intra-group analysis comparing baseline (T0) and final assessment (T1) demonstrated significant improvements in functional disability across all three groups, as assessed by the Wilcoxon signed-rank test for paired samples. In the PG group, the test indicated a pronounced reduction in RMDQ scores (Z = −4.541, *p* < 0.001), suggesting a consistent improvement in lumbar function at T1. The BG group also showed a statistically significant improvement (Z = −2.218, *p* = 0.027), supporting the effectiveness of the applied intervention. In the CG group, although the magnitude of the test statistic was smaller (Z = −2.401), the difference remained statistically significant (*p* = 0.016), indicating a relevant change between the two time points. Overall, these findings support the conclusion that all groups experienced a significant reduction in functional disability, with variations in the magnitude of therapeutic response, reflecting a general trend toward improved lumbar functional capacity (Table 4).

The Kruskal–Wallis analysis indicated significant differences among the three groups regarding the distribution of ODI scores at T0 (H = 10.047, df = 2, *p* = 0.007). Therefore, the null hypothesis that the distributions were similar was rejected. Post hoc tests with Bonferroni correction confirmed the presence of significant between-group differences. A significant difference was observed between CG and PG (test statistic = 16.897, Z = 2.584, p_adj_. = 0.029), while the difference between CG and BG was even more pronounced (test statistic = 19.693, Z = 2.942, p_adj._ = 0.010). The PG–BG comparison did not reveal a significant difference (test statistic = −2.796, Z = −0.441, p_adj._ = 1.000). These findings suggest that the level of functional disability, as measured by the ODI at baseline (T0), differed significantly depending on group allocation, particularly in comparisons involving the CG group. The Kruskal–Wallis analysis also revealed significant differences among the three groups in the distribution of ODI scores at T1 (H = 10.459, df = 2, *p* = 0.005). Consequently, the null hypothesis of similar distributions was rejected. Post hoc tests with Bonferroni correction identified a significant difference between CG and BG (test statistic = 21.566, Z = 3.220, p_adj._ = 0.004). A moderate difference was observed between CG and PG (test statistic = 9.732, Z = 1.487); however, the p_adj._ value (0.411) did not reach statistical significance. The PG–BG comparison did not indicate a significant difference (test statistic = −11.834, Z = −1.865, p_adj._ = 0.187). These results suggest that functional disability at T1 differed significantly between groups, particularly in the CG–BG comparison, where the difference was statistically robust. The remaining pairwise comparisons were not statistically significant after adjustment for multiple testing. The intra-group analysis comparing baseline (T0) and final assessment (T1) demonstrated significant improvements in lumbar mobility in two of the three groups, as assessed by the Wilcoxon signed-rank test for paired samples. In the PG group, the test indicated a significant increase in Schober scores (Z = −4.062, *p* < 0.001), suggesting a consistent improvement in lumbar flexibility at T1. The CG group also showed a statistically significant improvement (Z = −2.988, *p* = 0.003), indicating a relevant change between the two time points. In contrast, in the BG group, the observed change did not reach statistical significance (Z = −1.910, *p* = 0.056), suggesting a trend toward improvement without statistical confirmation. These findings indicate that the interventions or specific conditions applied in the PG and CG groups were associated with significant improvements in lumbar mobility, whereas in the BG group, the observed change remained statistically nonsignificant (Table 4).

The Kruskal–Wallis analysis indicated significant differences among the three groups regarding the distribution of Schober test scores at T0 (H = 30.680, df = 2, *p* < 0.001). Post hoc tests with Bonferroni correction confirmed that all pairwise group comparisons were statistically significant. Between BG and PG, the test statistic was 20.090, with a standardized Z value of 3.162 and an p_adj._-value of 0.005. The difference between BG and CG was more pronounced (test statistic = −37.009, Z = −5.518, p_adj._ < 0.001). A significant difference was also observed between PG and CG (test statistic = −19.919, Z = −2.582, p_adj._ = 0.029). The nonparametric analysis applied to Schober scores at T1 also revealed significant differences among the three groups (BG, PG, CG), according to the Kruskal–Wallis test (H = 16.130, df = 2, *p* < 0.001). Post hoc tests with Bonferroni correction showed that the difference between BG and CG was statistically significant (test statistic = −26.905, Z = −4.011, p_adj._ < 0.001), indicating a clear variation between these groups. The PG–CG comparison yielded a test statistic of −13.038, Z = −1.989, with an p_adj._ = 0.140, while the BG–PG comparison showed a test statistic of 13.867, Z = 2.182, p_adj._ = 0.087; both comparisons were not statistically significant after correction. Thus, only the BG–CG difference remained significant at T1. The intra-group analysis comparing baseline (T0) and final assessment (T1) demonstrated significant improvements across all three groups, as assessed by the Wilcoxon signed-rank test for paired samples. In the PG group, the test indicated a significant difference (Z = 4.813, *p* < 0.001), suggesting an improvement in Schober scores at T1. The BG group also showed a statistically significant improvement (Z = 4.574, *p* < 0.001), confirming the effectiveness of the applied intervention. In the CG group, although the magnitude of the test statistic was smaller (Z = 3.307), the difference remained statistically significant (*p* < 0.001), indicating a relevant change between the two time points, with improved Schober scores at T1. These findings support the conclusion that all groups experienced significant changes in Schober scores, with variations in the magnitude of therapeutic response (Table 5).

The Kruskal–Wallis test did not reveal significant differences among the three groups regarding the distribution of EQ-5D-5L scores at T0 (H = 3.624, df = 2, *p* = 0.163). Therefore, the null hypothesis that the distributions were similar was retained. The Kruskal–Wallis analysis indicated significant differences among the three groups in the distribution of EQ-5D-5L scores at T1 (H = 10.718, df = 2, *p* = 0.005). Post hoc tests with Bonferroni correction identified a significant difference between the BG and PG groups (test statistic = 20.210, standardized Z = 3.196, p_adj_. = 0.004). A marginal difference was observed between BG and CG (test statistic = −14.719, Z = −2.206); however, the padj-value (0.082) did not reach the threshold for statistical significance. The CG–PG comparison did not indicate significant differences (test statistic = 5.491, Z = 0.842, p_adj._ = 0.998). These results suggest that changes in EQ-5D-5L scores at T1 were significantly influenced by group allocation, particularly in the BG–PG comparison, where the difference was statistically robust. The intra-group analysis comparing baseline (T0) and final assessment (T1) demonstrated significant improvements across all three groups, as assessed by the Wilcoxon signed-rank test for paired samples. In the PG group, the test indicated a significant difference (Z = 4.262, *p* < 0.001), suggesting a relevant improvement in EQ-5D-5L scores at T1. The BG group also showed a statistically significant improvement (Z = 2.416, *p* = 0.016), supporting the effectiveness of the applied intervention. In the CG group, although the magnitude of the test statistic was smaller (Z = 2.823), the difference remained statistically significant (*p* = 0.005), indicating a meaningful change between the two time points, with improved quality-of-life scores at T1. These findings support the conclusion that all groups experienced significant changes in EQ-5D-5L scores, with variations in the magnitude of therapeutic response, reflecting a general trend toward improved perceived health status (Table 5).

## 4. Discussion

LBP remains a major public health concern, frequently leading to persistent pain, functional impairment, and reduced quality of life despite diverse therapeutic approaches [58]. Given its multidimensional nature, comprehensive assessment and well-designed clinical studies are essential to evaluate treatment effects across subjective and objective outcomes [59].

Balneotherapy is widely used for musculoskeletal disorders, including LBP; however, evidence remains heterogeneous. While some randomized trials report improvements in pain, function, and quality of life—particularly when combined with physiotherapy—methodological limitations and variability in study designs restrict definitive conclusions regarding its efficacy [60].

Prolotherapy has also been investigated as an interventional option for LBP. Although certain trials and reviews suggest potential benefits, especially when used adjunctively, the overall evidence remains inconclusive due to heterogeneity in protocols and frequent co-interventions, and its standalone effectiveness is not yet firmly established [28].

Against this background, the present open intervention study was conducted to assess the efficacy of commonly used therapeutic approaches for LBP, including balneotherapy-like modalities and structured treatment plans, with a focus on both subjective pain perception and objective pain sensitivity, as well as psychological outcomes, functional disability, mobility, and quality of life. In the present study, baseline demographic characteristics were largely comparable between the study groups, with the exception of age. Patients allocated to the balneotherapy plus physiotherapy group were significantly older than those in the control and prolotherapy groups. This finding is consistent with previous reports showing that balneotherapy-based interventions are more commonly applied in older patients with chronic musculoskeletal and degenerative conditions, due to their non-invasive nature and favorable safety profile [61,62].

The observed age difference likely reflects routine clinical practice rather than a selection bias. The older mean age observed in the balneotherapy group may have influenced functional recovery and mobility outcomes, as age-related factors such as reduced muscle strength, comorbidities, and longer disease duration can affect treatment response [63]. Therefore, comparative findings between groups should be interpreted cautiously. Spa-based and rehabilitation-oriented therapies are frequently recommended for elderly patients. In contrast, prolotherapy is more often used in younger or middle-aged individuals with higher functional demands and fewer comorbidities [26,33]. Nevertheless, this age imbalance should be considered when interpreting treatment outcomes, given the potential influence of age on pain perception, tissue healing, and response to rehabilitation [64].

Sex distribution was similar across groups, with a predominance of female participants. This pattern aligns with epidemiological data indicating a higher prevalence of chronic musculoskeletal pain and increased healthcare utilization among women [65]. The lack of significant differences in sex distribution minimizes the risk of sex-related confounding effects [66].

No significant differences were observed regarding body mass index categories, living environment, or physical activity level. The high prevalence of overweight and obesity across all groups is consistent with prior studies in chronic pain populations and reflects the well-documented association between excess body weight and musculoskeletal disorders [67,68]. Comparable physical activity levels and living environments further support the baseline homogeneity of the groups [69,70].

Although demographic characteristics were broadly comparable between groups (except age), significant baseline differences were observed in key clinical variables—such as pain intensity, disability, and lumbar mobility. These baseline imbalances may have influenced subsequent changes over time and limited the extent to which post-intervention differences can be attributed solely to the applied interventions. Nevertheless, the general similarity in baseline demographic characteristics contributes to strengthening the internal validity of the study and supports the interpretation that observed outcome differences are less likely to be explained by demographic disparities alone [71].

Regarding pain outcomes, although both VAS and PPT are commonly used to assess pain, they reflect different dimensions of the pain experience and are not necessarily expected to show parallel changes [72]. In this study, while VAS scores significantly decreased across all groups, indicating an overall reduction in perceived pain intensity, changes in PPT values were more limited, with significant improvement observed only in the patient group on the left side. While pain intensity decreased significantly within all groups, no significant between-group differences were observed at T1 for VAS or PPT measures, indicating comparable short-term effects of the three interventions on pain perception and pressure pain sensitivity. Clinically, although a statistically significant difference after Bonferroni correction was observed only between BG and PG, caution is warranted when interpreting pain outcomes—statistical significance does not necessarily reflect meaningful improvement in patient-reported pain, and minimal clinically important differences should be considered alongside *p*-values when assessing the real impact of treatments on pain [73]. This partial dissociation suggests that the subjective experience of pain (as captured by VAS) may improve due to factors such as psychological relief, expectation, or general well-being, even when objective measures of PPT remain unchanged [74]. Conversely, physiological adaptations in pain threshold may not always be accompanied by perceived reductions in pain. These findings underscore the importance of using both subjective and objective tools when evaluating pain, as they may capture distinct yet complementary aspects of pain modulation [75].

Despite significant within-group reductions in depressive symptom scores—particularly in PG and CG for HADS-D and in all groups for PHQ-9 and BDI-II—no significant between-group differences were observed at T1, suggesting comparable effects of the three interventions on mood-related outcomes. In the present cohort, improvements in psychological outcomes paralleled changes in clinical pain and functional status, consistent with evidence of high rates of depression and anxiety among patients with chronic musculoskeletal pain (~40% prevalence) [76]. Patients with greater reductions in pain intensity and functional limitations often report concurrent improvements in anxiety and depressive symptoms, reflecting the close interdependence between physical and psychological domains in chronic pain [77,78].

Thermal-water interventions such as balneotherapy and hydrotherapy have been associated with significant reductions in anxiety and depression in adults with musculoskeletal and stress-related symptoms, suggesting that somatic and psychological effects may be mutually reinforcing [79,80]. Meta-analytic evidence supports the effectiveness of these interventions in alleviating psychological distress alongside pain and disability, with improvements that may persist beyond the immediate treatment period [79,81].

The lack of significant between-group differences in psychological outcomes echoes findings that non-specific therapeutic factors (e.g., structured follow-up, patient engagement) contribute to mental health improvements across diverse intervention types. Nevertheless, the observed psychological changes correlate with the magnitude of pain reduction and functional improvement, underscoring that interventions targeting physical symptoms can indirectly enhance emotional well-being in chronic pain populations [6,82]. Among the psychological measures, PHQ-9 demonstrated significant reductions across all three groups, suggesting a consistent decrease in depressive symptom burden. PHQ-9 captures core depressive domains such as anhedonia, low mood, sleep disturbance, fatigue, and concentration difficulties, which are frequently exacerbated in LBP [53]. The observed improvement may reflect not only pain reduction but also enhanced functional capacity and perceived self-efficacy following intervention. Given the bidirectional relationship between chronic pain and depressive symptoms, even modest improvements in physical status may contribute to meaningful changes in affective well-being. However, the absence of significant between-group differences indicates that these psychological benefits were not specific to a single intervention but rather represent a general therapeutic effect associated with structured treatment and follow-up [53].

In our study, disability outcomes assessed by the RMDQ and the ODI improved significantly over time, with the most pronounced effects observed in the prolotherapy group. Both within- and between-group analyses indicated greater reductions in disability scores following prolotherapy compared with balneotherapy and control interventions [21].

The greater functional improvement associated with prolotherapy may be explained by its targeted biomechanical mechanism, aimed at reducing nociceptive input and improving segmental stability. Recent evidence suggests that interventions addressing structural and functional contributors to LBP are more likely to yield clinically meaningful reductions in disability [32,83,84].

The observed disability improvements in the prolotherapy group paralleled reductions in pain intensity and psychological distress, supporting the concept that functional recovery in chronic musculoskeletal pain reflects the interaction between physical and psychosocial factors [85,86]. In contrast, the more modest disability changes observed in the balneotherapy group may be partially attributed to older age and longer symptom duration, factors known to limit functional recovery despite symptom relief [87,88].

Both within- and between-group analyses indicated statistically significant differences, with prolotherapy showing a more consistent improvement in lumbar mobility compared to balneotherapy and control interventions.

Significant improvements in spinal mobility, assessed by the Schober test, were observed across all groups, with the greatest magnitude of change in the prolotherapy group. Both within- and between-group analyses demonstrated statistically significant differences, with prolotherapy showing a more consistent improvement in lumbar spine mobility compared to balneotherapy and control interventions. These findings are consistent with recent evidence indicating that interventional treatments targeting ligamentous and segmental dysfunction can lead to meaningful improvements in spinal mobility by reducing pain-related movement inhibition and enhancing functional stability. Improvements in mobility are clinically relevant, as restricted lumbar flexion is a key determinant of functional limitation in LBP [69,85].

Quality of life, as measured by the EQ-5D-5L, improved significantly across all groups. However, between-group comparisons favored prolotherapy at follow-up, indicating a greater overall health-related quality-of-life benefit. This pattern suggests that improvements in mobility and pain may translate into broader gains in perceived health status, particularly for interventions that achieve superior functional outcomes [89].

The parallel improvements observed in mobility, disability, pain intensity, and psychological measures support a multidimensional treatment effect, in line with contemporary biopsychosocial models of LBP. In contrast, although balneotherapy produced significant within-group improvements in mobility and quality of life, the absence of consistent intergroup superiority may reflect age-related factors and longer symptom chronicity in this cohort, which are known to limit functional adaptation [88]. From a clinical perspective, the absence of significant baseline differences (T0) in EQ-5D-5L scores suggests initial comparability between groups in terms of perceived health status, strengthening the internal validity of post-intervention comparisons. At T1, however, quality-of-life outcomes differed significantly between groups, particularly in the BG–PG comparison, indicating that group allocation influenced perceived health improvements. The EQ-5D-5L is a widely validated generic measure of health-related quality of life (HRQoL), sensitive to changes in patients with musculoskeletal disorders, including low back pain [90]. Improvements observed across all groups are consistent with evidence that multidisciplinary or structured therapeutic interventions can positively influence both functional status and perceived health [91].

Given the multifactorial nature of musculoskeletal pain and the demonstrated efficacy of multidisciplinary therapeutic approaches—including physiotherapy and targeted local interventions—such strategies may serve as valuable components within an integrative pain management framework [92].

Effective pain management requires a biopsychosocial approach that addresses not only biological factors but also the psychological and social dimensions of the patient—an essential perspective in contemporary rehabilitation medicine [93].

### 4.1. Study Strengths

A major strength of this study is the multidimensional evaluation of treatment effects, encompassing pain intensity, psychological outcomes, disability, spinal mobility, and health-related quality of life. This comprehensive approach is consistent with current recommendations for LBP assessment, which emphasize the integration of physical and psychosocial domains. Another strength is the comparative design, including prolotherapy, balneotherapy plus physiotherapy, and control groups, allowing evaluation of commonly used therapeutic strategies in routine clinical practice. The use of validated and widely accepted outcome measures (HADS, PHQ-9, BDI-II, RMDQ, ODI, Schober test, and EQ-5D-5L) enhances the reliability of the findings.

### 4.2. Study Limitations

Several limitations should be acknowledged. First, the relatively small sample size in each group may have limited the statistical power to detect subtle between-group differences. Second, the study groups were not fully age-matched, with older patients in the balneotherapy plus physiotherapy group. Third, the follow-up period was limited to short-term assessment, preventing evaluation of the long-term sustainability of treatment effects. Additionally, the use of self-reported questionnaires may introduce response bias, and objective functional measures could strengthen future research. Also, baseline differences in pain, disability, and lumbar mobility were observed between groups. These imbalances, likely related to the non-randomized design of the study, represent an important methodological limitation. The absence of randomization may have introduced selection bias and reduced group comparability at baseline, potentially influencing follow-up changes. Furthermore, the statistical approach, based primarily on non-parametric within- and between-group comparisons, did not formally assess group × time interaction effects, which limits the ability to draw definitive conclusions regarding treatment superiority. In the end, the lack of blinding and the potential influence of non-specific treatment effects cannot be excluded.

### 4.3. Future Research Directions

Future studies should include larger, age-matched cohorts and longer follow-up periods to evaluate the durability of treatment effects. Randomized controlled designs with objective functional measures and stratification by baseline clinical and psychological characteristics may further clarify the comparative effectiveness of prolotherapy and balneotherapy. Future research should include more robust statistical analyses and more rigorous methodological structuring. Additionally, exploring combined or sequential therapeutic approaches could provide insight into optimized management strategies for LBP.

## 5. Conclusions

All evaluated interventions improved clinical, functional, and psychological outcomes in patients with LBP. Prolotherapy showed a pattern of more consistent improvements, particularly in disability, spinal mobility, and health-related quality of life. These findings should be interpreted cautiously, given the non-randomized design and baseline differences between groups. Further randomized studies with larger samples and longer follow-up are warranted to confirm these results.

## Figures and Tables

**Table 1:** Baseline demographic characteristics of the patients.

Variable	CG (n = 25)	BG (n = 28)	PG (n = 31)	*p*-Value
Age (years), mean ± SD	48.28 ± 14.04	63.29 ± 9.49	48.77 ± 14.23	<0.001
Sex, female, n (%)	17 (68.0)	19 (67.9)	24 (77.4)	0.673
Body mass index category, n (%)				0.112
Normal weight	11 (44.0)	7 (25.0)	14 (45.2)	
Overweight	7 (28.0)	15 (53.6)	14 (45.2)	
Grade I obesity	7 (28.0)	6 (21.4)	3 (9.7)	
Living environment, urban, n (%)	18 (72.0)	24 (85.7)	24 (77.4)	0.401
Physical activity level, n (%)				0.537
Sedentary	5 (20.0)	8 (28.6)	6 (19.4)	
Moderate	9 (36.0)	15 (53.6)	13 (41.9)	
Active	11 (44.0)	5 (17.9)	12 (38.7)	

**Legend:** CG = Control Group; BG = Balneotherapy plus Physiotherapy Group; PG = Prolotherapy Group; SD = Standard Deviation; *p* = *p*-value.

**Table 2:** Pain Outcomes at T0 and T1: Subjective (VAS) and Objective (PPT) Measures.

The Analyzed Parameter	Group	Wilcoxon Test		Kruskal–Wallis Test					
				T0			T1		
		Z	*p*	H	df	*p*	H	df	*p*
**VAS**	PG	−4.880	<0.001	8.442	2	0.015	0.595	2	0.743
	BG	−3.990	<0.001						
	CG	−4.315	<0.001						
**PPT RS**	PG	0.803	0.422	1.197	2	0.550	0.896	2	0.639
	BG	0.023	0.982						
	CG	−1.184	0.236						
**PPT LS**	PG	2.273	0.023	2.155	2	0.341	1.241	2	0.538
	BG	0.068	0.946						
	CG	0.632	0.527						
**TOTAL PPT**	PG	1.254	0.210	0.998	2	0.607	0.019	2	0.991
	BG	0.319	0.750						
	CG	−0.498	0.619						

**Legend:** VAS = Visual Analog Scale; PPT = Pressure Pain Threshold; PG = Prolotherapy Group; BG = Balneotherapy plus Physiotherapy Group; CG = Control Group; RS = Right Side; LS = Left Side; T0 = Baseline; T1 = 4-Week Follow-Up; Z = Wilcoxon Signed-Rank Test statistic; H = Kruskal–Wallis Test statistic; df = Degrees of Freedom; *p* = *p*-value. Within-group comparisons (T0 vs. T1) were performed using the Wilcoxon signed-rank test. Between-group comparisons at T0 and T1 were conducted using the Kruskal–Wallis test.

**Table 3:** Psychological Outcomes at T0 and T1.

The Analyzed Parameter	Group	Wilcoxon Test		Kruskal–Wallis Test					
				T0			T1		
		Z	*p*	H	df	*p*	H	df	*p*
**HADS-A**	PG	−2.207	0.027	3.611	2	0.164	4.179	2	0.124
	BG	−1.101	0.271						
	CG	−2.194	0.028						
**HADS-D**	PG	−2.716	0.007	2.614	2	0.271	3.164	2	0.206
	BG	−1.880	0.060						
	CG	−2.724	0.006						
**PHQ-9**	PG	−3.279	0.001	3.352	2	0.187	1.748	2	0.417
	BG	−3.126	0.002						
	CG	−2.530	0.011						
**BDI-II**	PG	−3.348	<0.001	2.862	2	0.239	2.685	2	0.261
	BG	−2.971	0.003						
	CG	−2.850	0.004						

**Legend:** HADS-A = Hospital Anxiety and Depression Scale—Anxiety; HADS-D = Hospital Anxiety and Depression Scale—Depression; PHQ-9 = Patient Health Questionnaire-9; BDI-II = Beck Depression Inventory-II; PG = Prolotherapy Group; BG = Balneotherapy plus Physiotherapy Group; CG = Control Group; T0 = Baseline; T1 = 4-Week Follow-Up; Z = Wilcoxon Signed-Rank Test statistic; H = Kruskal–Wallis Test statistic; df = Degrees of Freedom; *p* = *p*-value. Within-group comparisons (T0 vs. T1) were performed using the Wilcoxon signed-rank test. Between-group comparisons at T0 and T1 were conducted using the Kruskal–Wallis test.

**Table 4:** Disability Outcomes at T0 and T1.

The Analyzed Parameter	Group	Wilcoxon Test		Kruskal–Wallis Test					
				T0			T1		
		Z	*p*	H	df	*p*	H	df	*p*
**RMDQ**	PG	−4.541	<0.001	10.339	2	0.006	7.900	2	0.019
	BG	−2.218	0.027						
	CG	−2.401	0.016						
**ODI**	PG	−4.062	0.001	10.047	2	0.007	10.459	2	0.005
	BG	−1.910	0.056						
	CG	−2.988	0.003						

Legend: RMDQ = Roland-Morris Disability Questionnaire; ODI = Oswestry Disability Index; PG = Prolotherapy Group; BG = Balneotherapy plus Physiotherapy Group; CG = Control Group; T0 = Baseline; T1 = 4-Week Follow-Up; Z = Wilcoxon Signed-Rank Test statistic; H = Kruskal–Wallis Test statistic; df = Degrees of Freedom; *p* = *p*-value. Within-group comparisons (T0 vs. T1) were performed using the Wilcoxon signed-rank test. Between-group comparisons at T0 and T1 were conducted using the Kruskal–Wallis test.

**Table 5:** Mobility and Quality of Life Outcomes at T0 and T1.

The Analyzed Parameter	Group	Wilcoxon Test		Kruskal–Wallis Test					
				T0			T1		
		Z	*p*	H	df	*p*	H	df	*p*
**Schober**	PG	4.813	<0.001	30.680	2	<0.001	16.130	2	<0.001
	BG	4.574	<0.001						
	CG	3.307	<0.001						
**EQ-5D-5L**	PG	4.262	<0.001	3.624	2	0.163	10.718	2	0.005
	BG	2.416	0.016						
	CG	2.823	0.005						

Legend: Schober = Modified Schober Test; EQ-5D-5L = EuroQol 5-Dimension 5-Level; PG = Prolotherapy Group; BG = Balneotherapy plus Physiotherapy Group; CG = Control Group; T0 = Baseline; T1 = 4-Week Follow-Up; Z = Wilcoxon Signed-Rank Test statistic; H = Kruskal–Wallis Test statistic; df = Degrees of Freedom; *p* = *p*-value. Within-group comparisons (T0 vs. T1) were performed using the Wilcoxon signed-rank test. Between-group comparisons at T0 and T1 were conducted using the Kruskal–Wallis test.

## Data Availability

The original contributions presented in this study are included in the article/Supplementary Material. Further inquiries can be directed to the corresponding author.

## Supplementary Materials

The following supporting information can be downloaded at: https://www.mdpi.com/article/10.3390/medicina62040623/s1, Ref. [94] is listed in the supplementary.

## Abbreviations

The following abbreviations are used in this manuscript:

BDI-II	Beck Depression Inventory-II
BG	Balneotherapy plus physiotherapy group
CG	Standard physiotherapy control group
DF	Degrees of Freedom
EQ-5D-5L	EuroQol 5-Dimension 5-Level questionnaire
H	Kruskal–Wallis Test statistic
HADS-A	Hospital Anxiety and Depression Scale—Anxiety
HADS-D	Hospital Anxiety and Depression Scale—Depression
LBP	Chronic low back pain
LS	Left Side
P	*p*-value
PG	Prolotherapy group
PHQ-9	Patient Health Questionnaire-9
PPT	Pressure pain threshold
ODI	Oswestry Disability Index
RMDQ	Roland–Morris Disability Questionnaire
RS	Right Side
SD	Standard deviation
T0	Baseline assessments
T1	Treatment completion
VAS	Visual Analog Scale
Z	Wilcoxon Signed-Rank Test statistic
